# Supplementary material for: Smaller climatic niche shifts in invasive than non-invasive alien ant species
Source: Nat Commun. 2020 Oct 15;11:5213. doi: 10.1038/s41467-020-19031-1 (PMC7567077; doi:10.1038/s41467-020-19031-1)
Supplement: Supplementary file 1 — Supplementary Information [file 41467_2020_19031_MOESM1_ESM.pdf]

Supplementary Information

Figures

Fig. S1. Distribution of native and non-native range niches for all 82 species. For each graph, the red distribution red (right) represents the non-native niche, and the blue (left) the native niche.

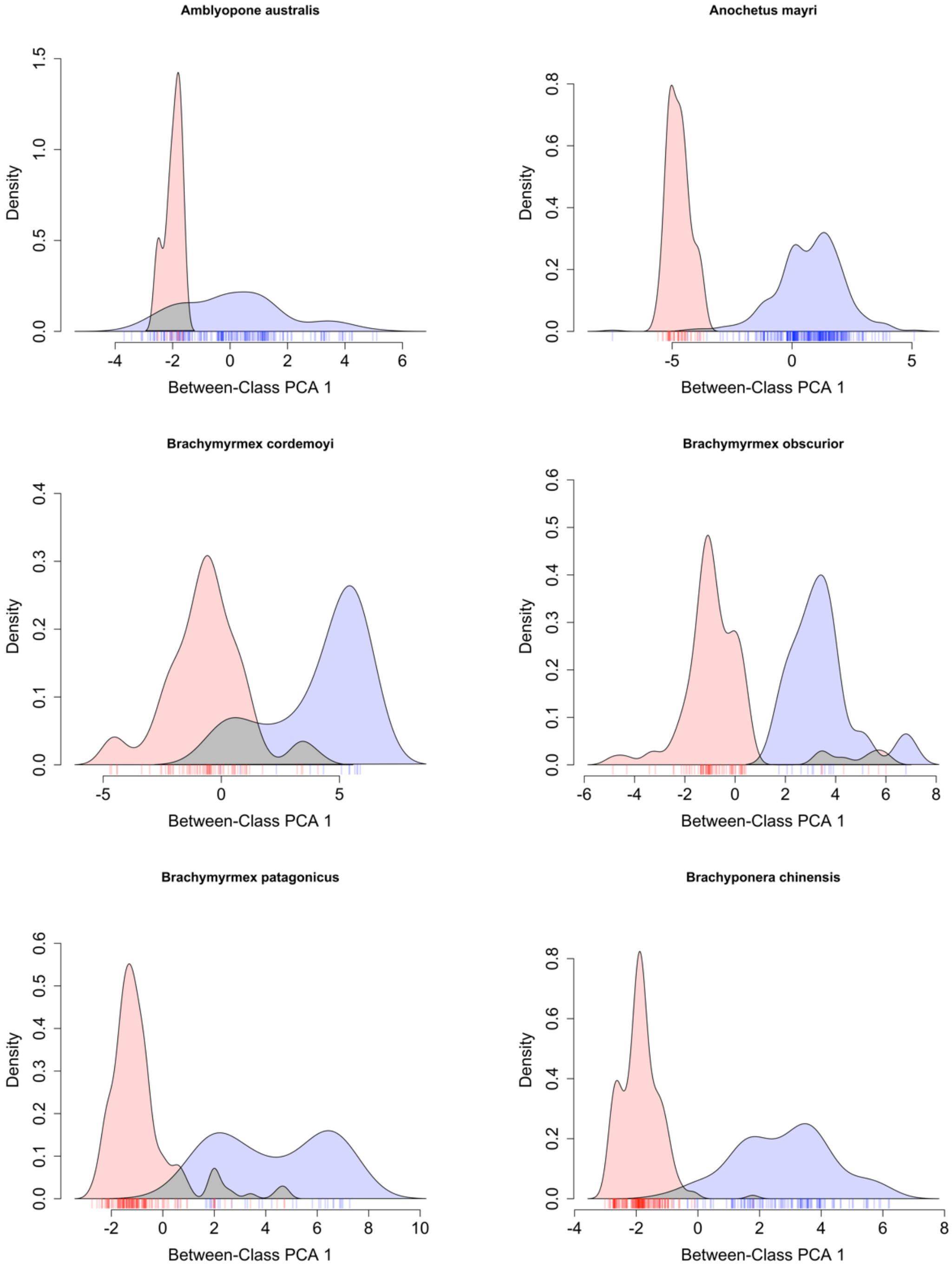
Amblyopone australis
Anochetus mayri
Brachymyrmex cordemoyi
Brachymyrmex obscurior
Brachymyrmex patagonicus
Brachyponera chinensis
Density
Between-Class PCA 1

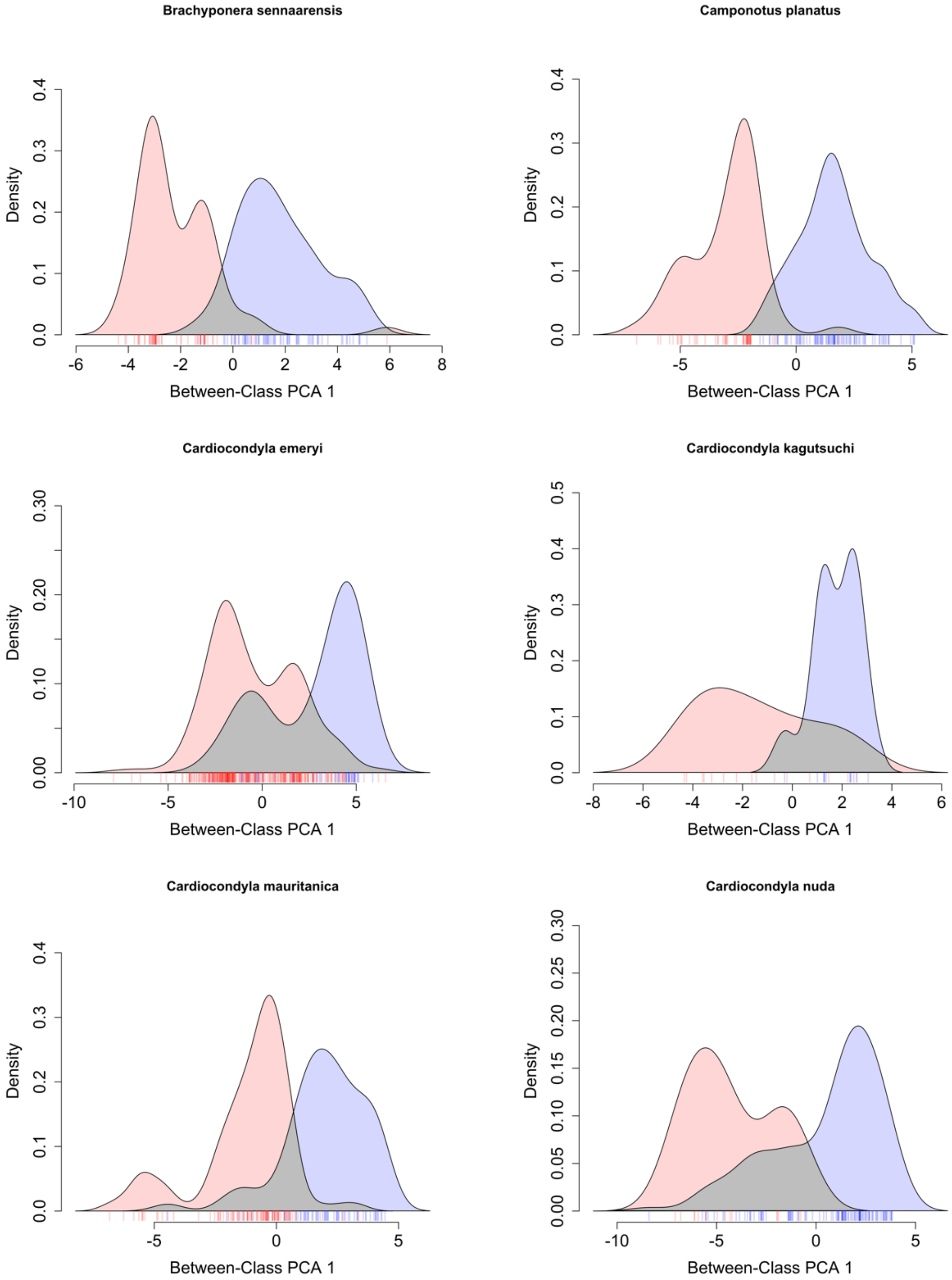
Brachyponera sennaarensis
Density
Between-Class PCA 1
Camponotus planatus
Density
Between-Class PCA 1
Cardiocondyla emeryi
Density
Between-Class PCA 1
Cardiocondyla kagutsuchi
Density
Between-Class PCA 1
Cardiocondyla mauritanica
Density
Between-Class PCA 1
Cardiocondyla nuda
Density
Between-Class PCA 1

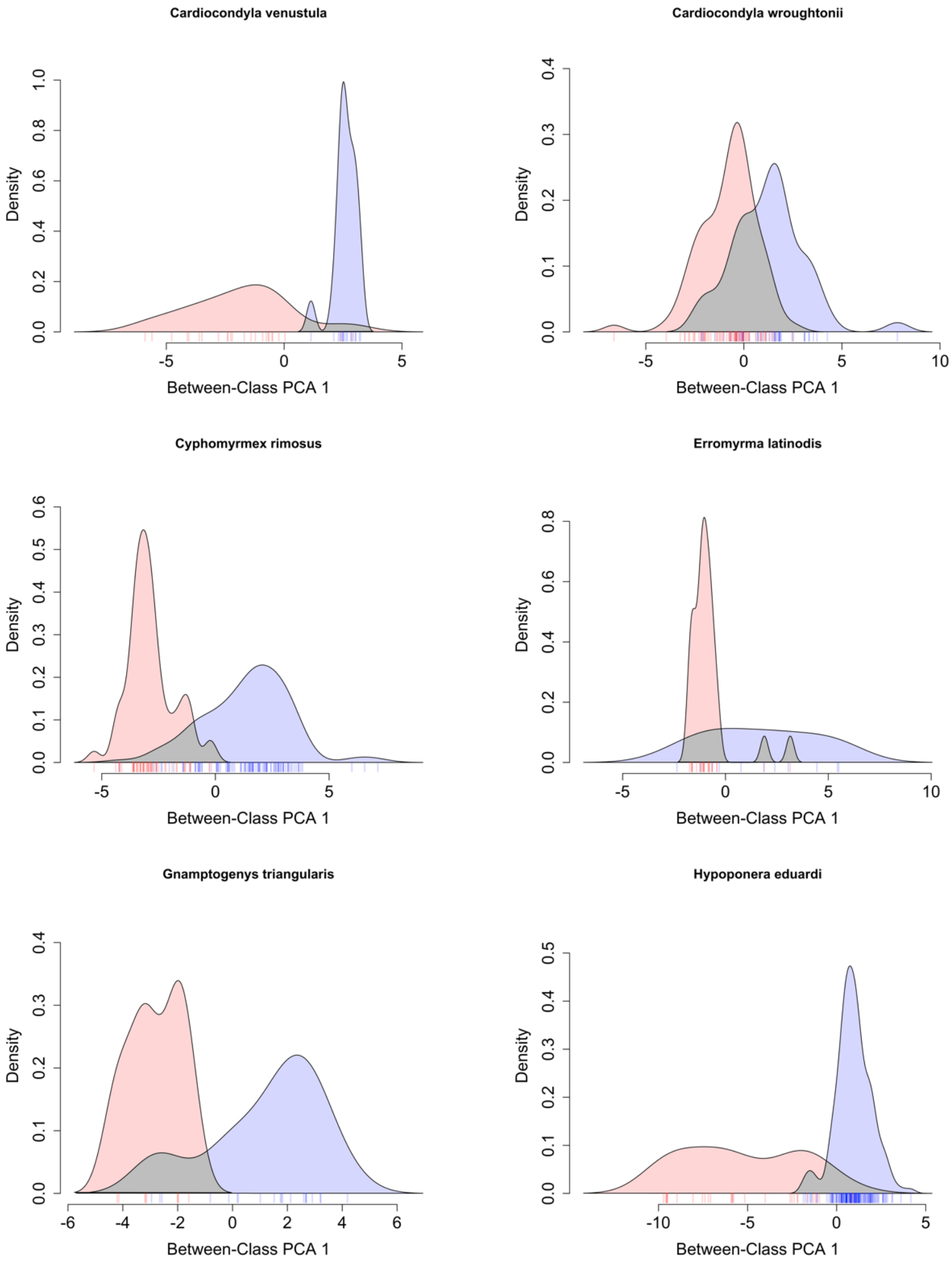
Cardiocondyla venustula
Cardiocondyla wroughtonii
Cyphomyrmex rimosus
Erromyrma latinodis
Gnamptogenys triangularis
Hypoponera eduardi
Density
Between-Class PCA 1

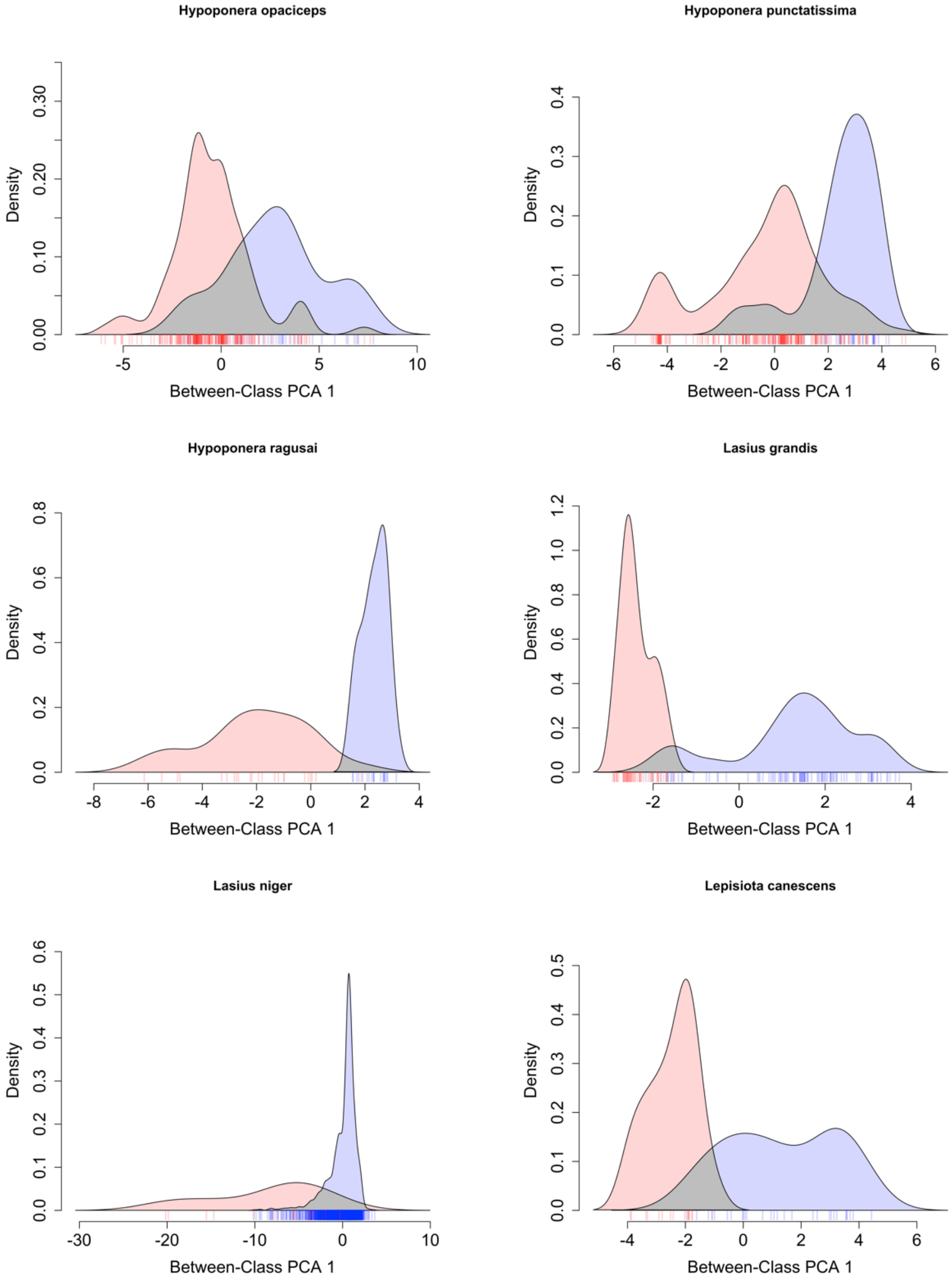
Hypoponera opaciceps
Density
0.00 0.10 0.20 0.30
-5 0 5 10
Between-Class PCA 1
Hypoponera punctatissima
Density
0.0 0.1 0.2 0.3 0.4
-6 -4 -2 0 2 4 6
Between-Class PCA 1
Hypoponera ragusai
Density
0.0 0.2 0.4 0.6 0.8
-8 -6 -4 -2 0 2 4
Between-Class PCA 1
Lasius grandis
Density
0.0 0.2 0.4 0.6 0.8 1.0 1.2
-2 0 2 4
Between-Class PCA 1
Lasius niger
Density
0.0 0.1 0.2 0.3 0.4 0.5 0.6
-30 -20 -10 0 10
Between-Class PCA 1
Lepisiota canescens
Density
0.0 0.1 0.2 0.3 0.4 0.5
-4 -2 0 2 4 6
Between-Class PCA 1

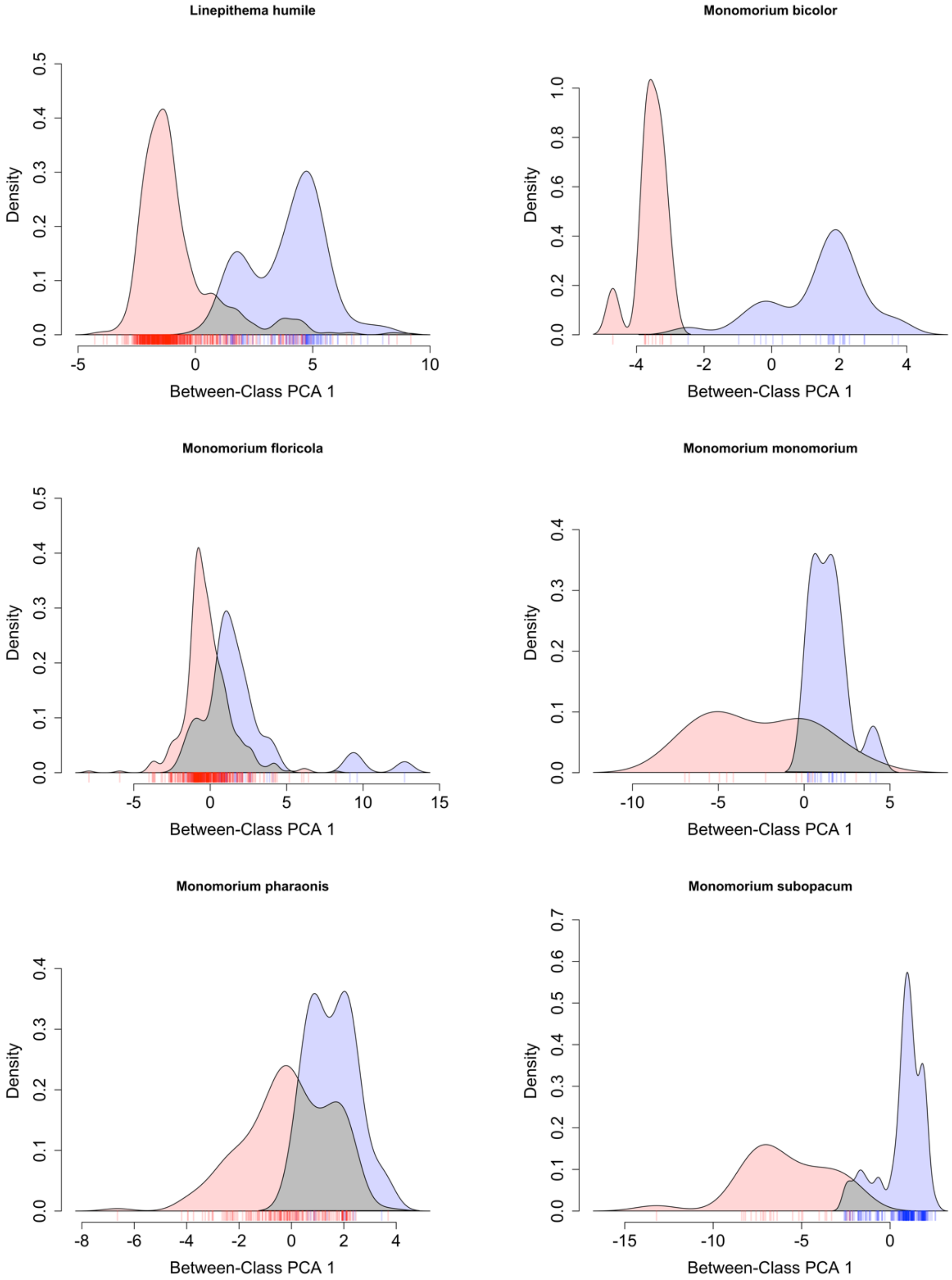
Linepithema humile
Monomorium bicolor
Monomorium floricola
Monomorium monomorium
Monomorium pharaonis
Monomorium subopacum
Density
Between-Class PCA 1

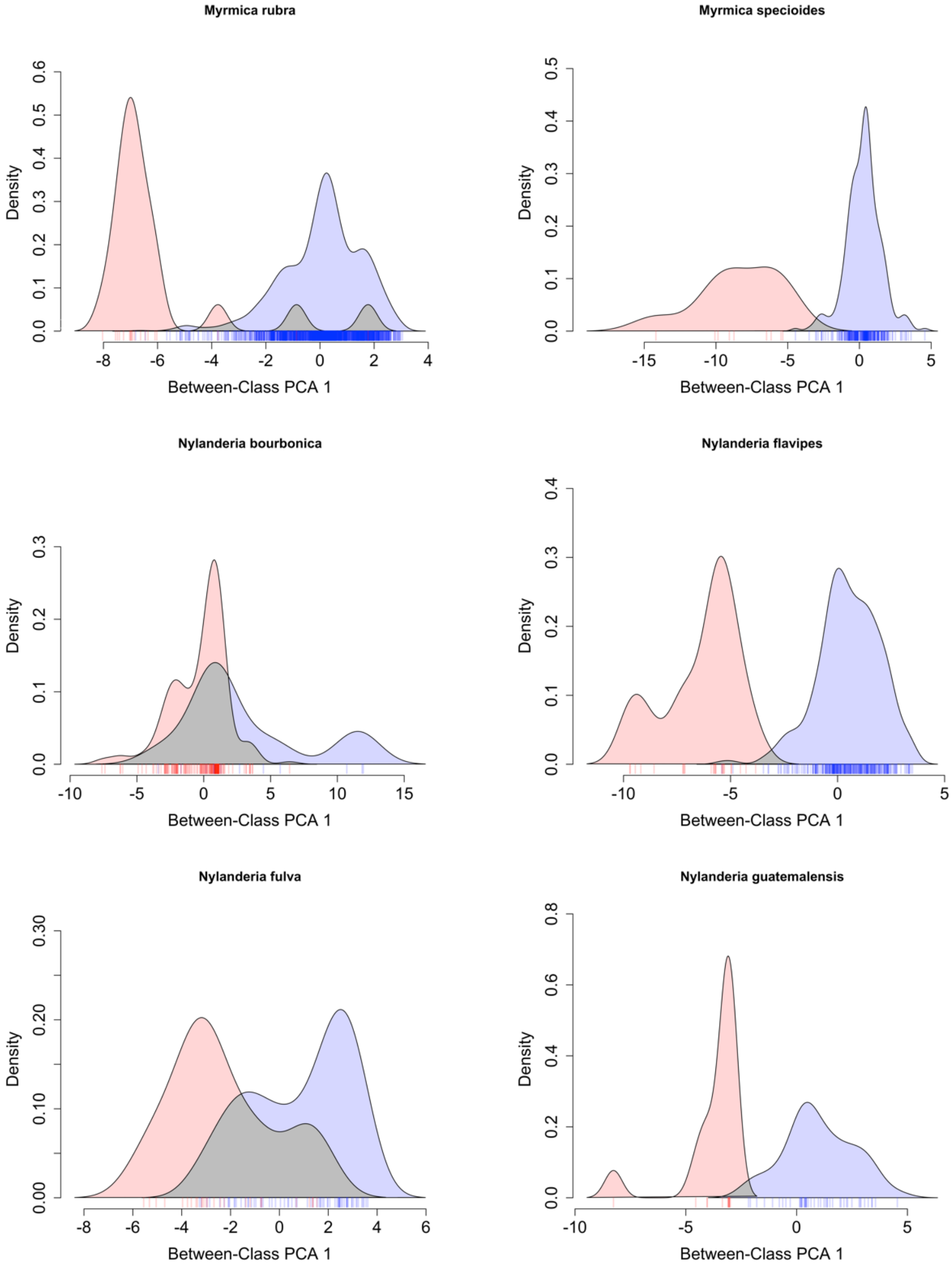
Myrmica rubra
Myrmica specioides
Nylanderia bourbonica
Nylanderia flavipes
Nylanderia fulva
Nylanderia guatemalensis
Density
Between-Class PCA 1

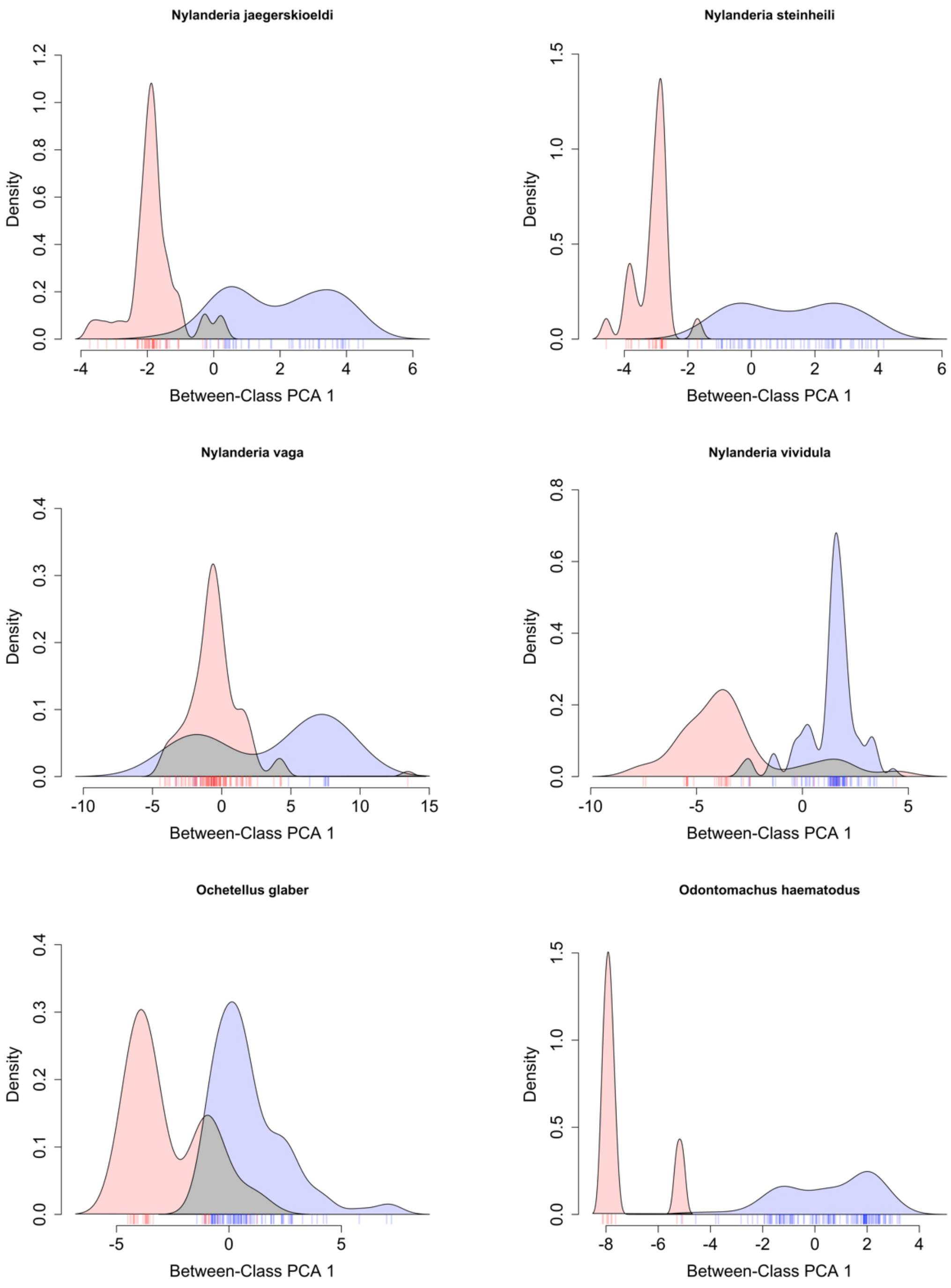
Nylanderia jaegerskioeldi
Density
Between-Class PCA 1
Nylanderia steinheili
Density
Between-Class PCA 1
Nylanderia vaga
Density
Between-Class PCA 1
Nylanderia vividula
Density
Between-Class PCA 1
Ochetellus glaber
Density
Between-Class PCA 1
Odontomachus haematodus
Density
Between-Class PCA 1

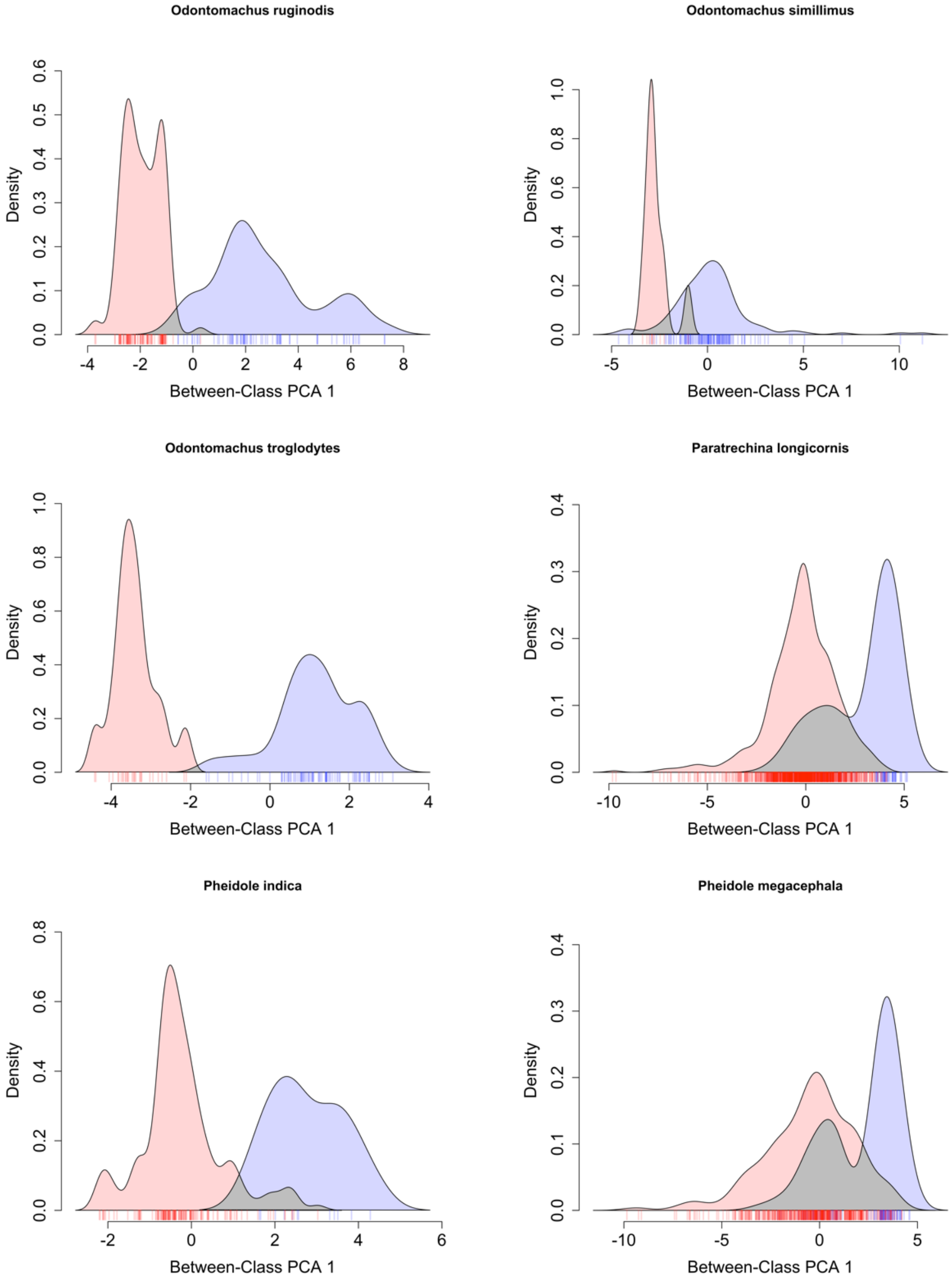
Odontomachus ruginodis
Density
Between-Class PCA 1
Odontomachus simillimus
Density
Between-Class PCA 1
Odontomachus troglodytes
Density
Between-Class PCA 1
Paratrechina longicornis
Density
Between-Class PCA 1
Pheidole indica
Density
Between-Class PCA 1
Pheidole megacephala
Density
Between-Class PCA 1

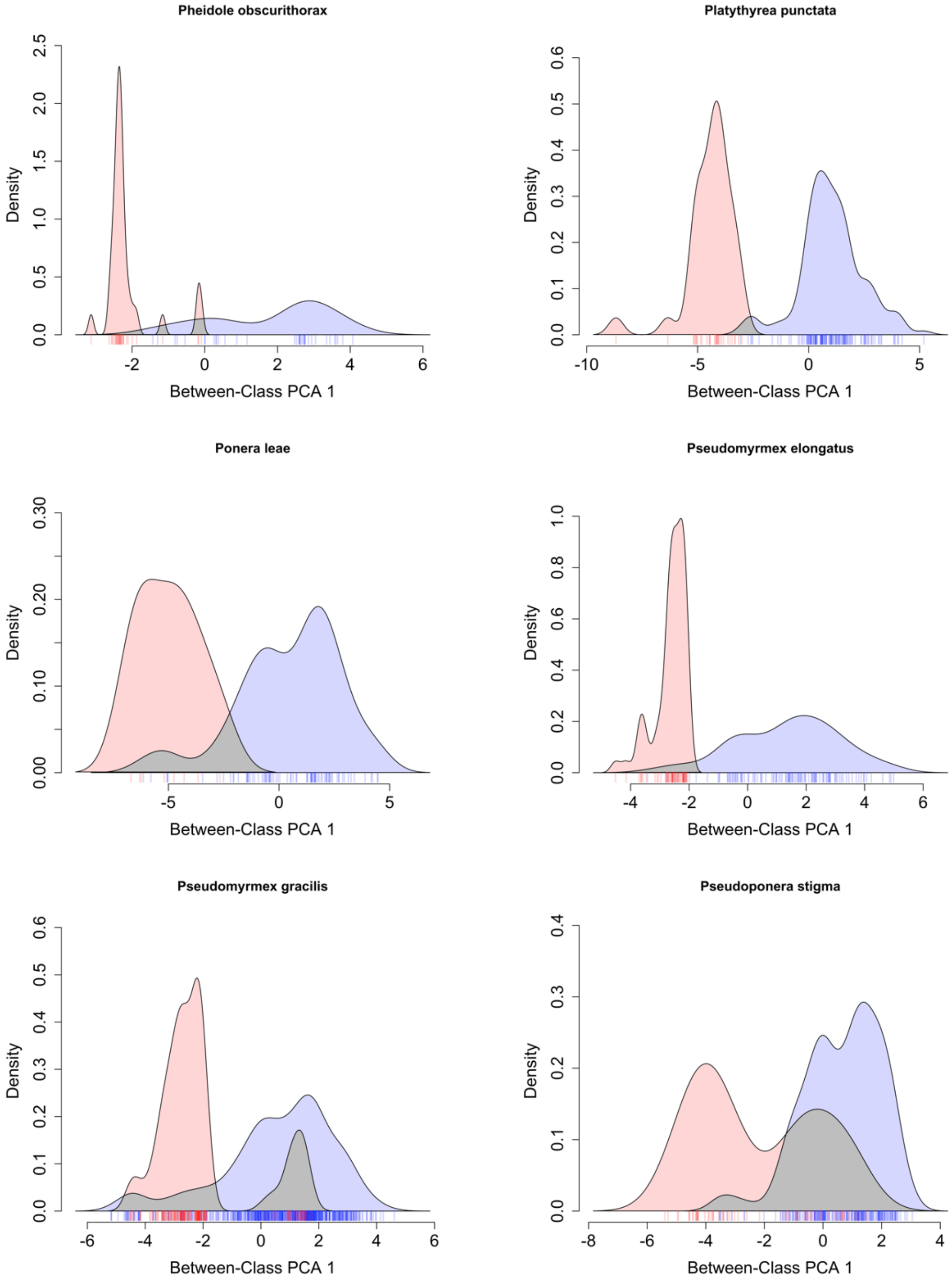
Pheidole obscurithorax
Density
0.0 0.5 1.0 1.5 2.0 2.5
-2 0 2 4 6
Between-Class PCA 1
Platythyrea punctata
Density
0.0 0.1 0.2 0.3 0.4 0.5 0.6
-10 -5 0 5
Between-Class PCA 1
Ponera leae
Density
0.00 0.10 0.20 0.30
-5 0 5
Between-Class PCA 1
Pseudomyrmex elongatus
Density
0.0 0.2 0.4 0.6 0.8 1.0
-4 -2 0 2 4 6
Between-Class PCA 1
Pseudomyrmex gracilis
Density
0.0 0.1 0.2 0.3 0.4 0.5 0.6
-6 -4 -2 0 2 4 6
Between-Class PCA 1
Pseudoponera stigma
Density
0.0 0.1 0.2 0.3 0.4
-8 -6 -4 -2 0 2 4
Between-Class PCA 1

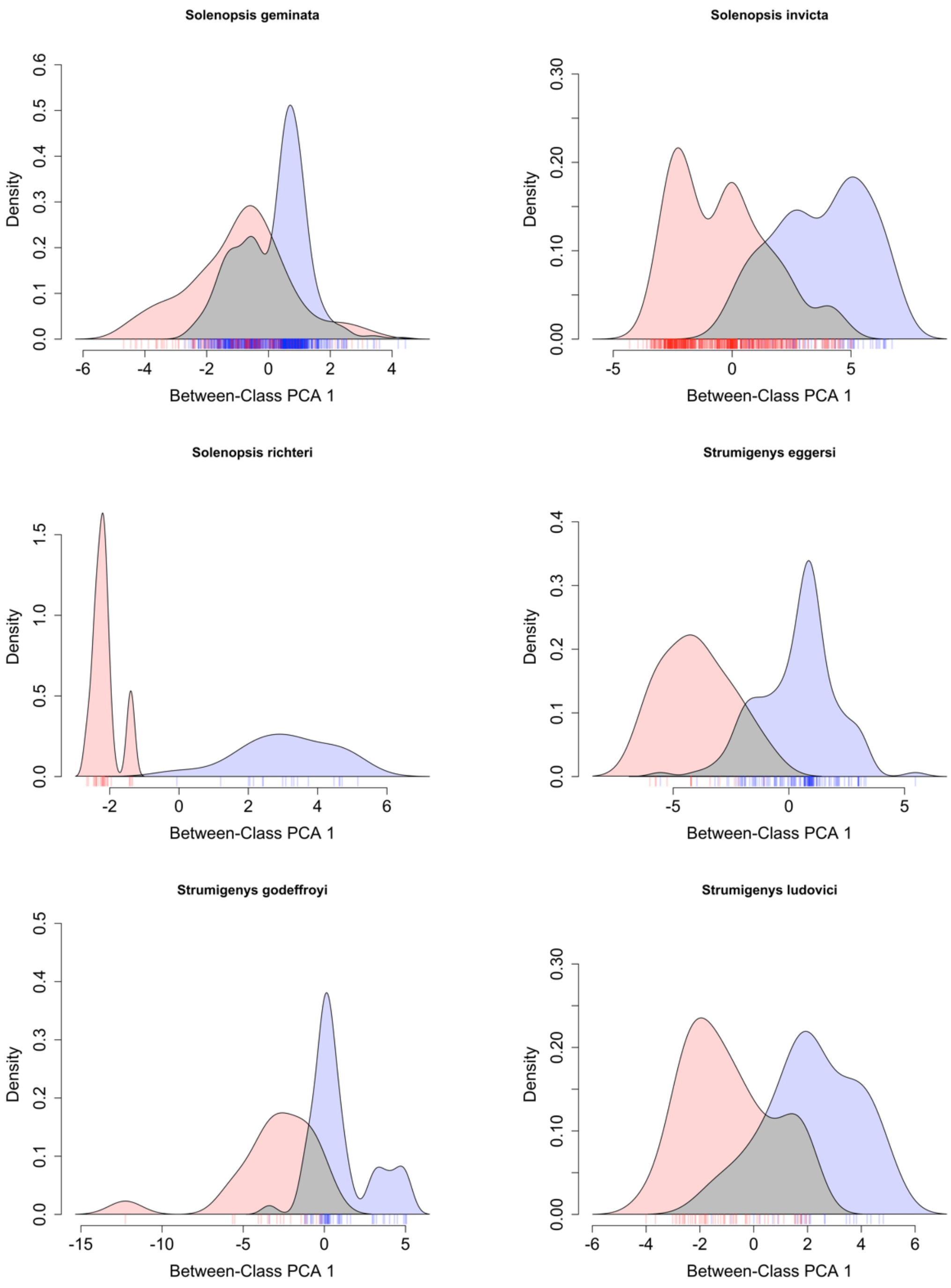
Solenopsis geminata
Density
Between-Class PCA 1
Solenopsis invicta
Density
Between-Class PCA 1
Solenopsis richteri
Density
Between-Class PCA 1
Strumigenys eggersi
Density
Between-Class PCA 1
Strumigenys godeffroyi
Density
Between-Class PCA 1
Strumigenys ludovici
Density
Between-Class PCA 1

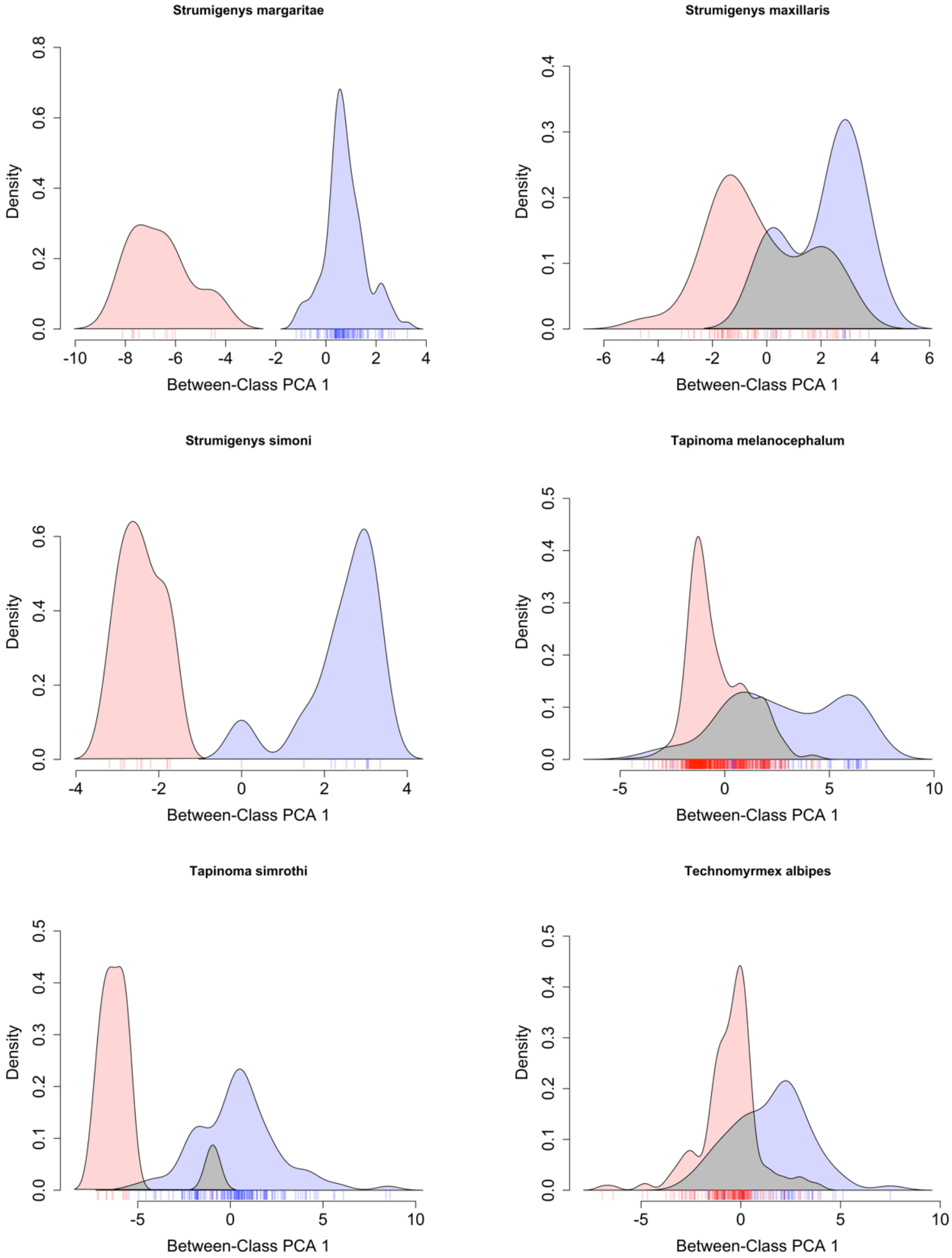

Strumigenys margaritae
Density
Between-Class PCA 1
Strumigenys maxillaris
Density
Between-Class PCA 1
Strumigenys simoni
Density
Between-Class PCA 1
Tapinoma melanocephalum
Density
Between-Class PCA 1
Tapinoma simrothi
Density
Between-Class PCA 1
Technomyrmex albipes
Density
Between-Class PCA 1

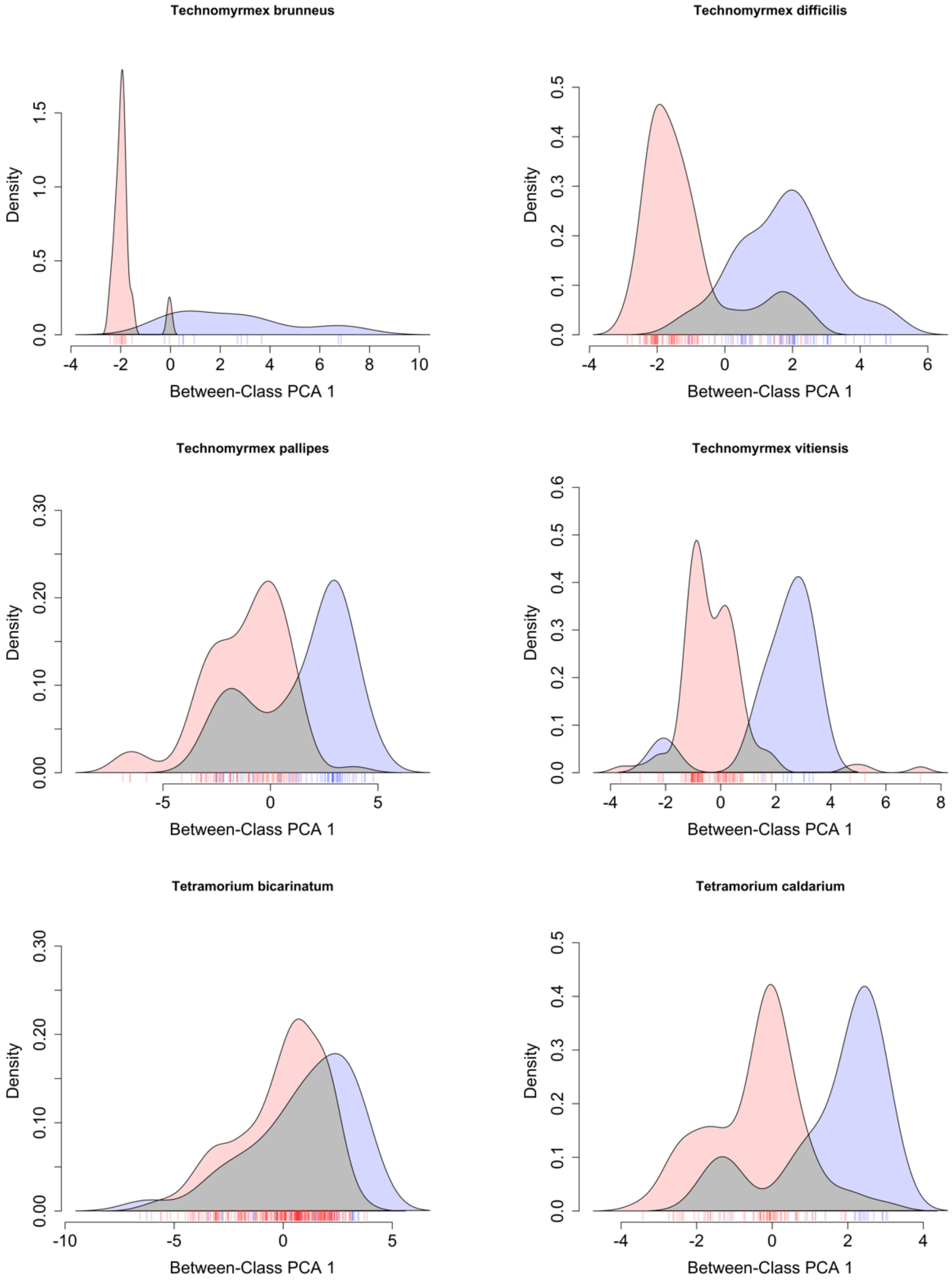

Technomyrmex brunneus
Technomyrmex difficilis
Technomyrmex pallipes
Technomyrmex vitiensis
Tetramorium bicarinatum
Tetramorium caldarium
Density
Between-Class PCA 1

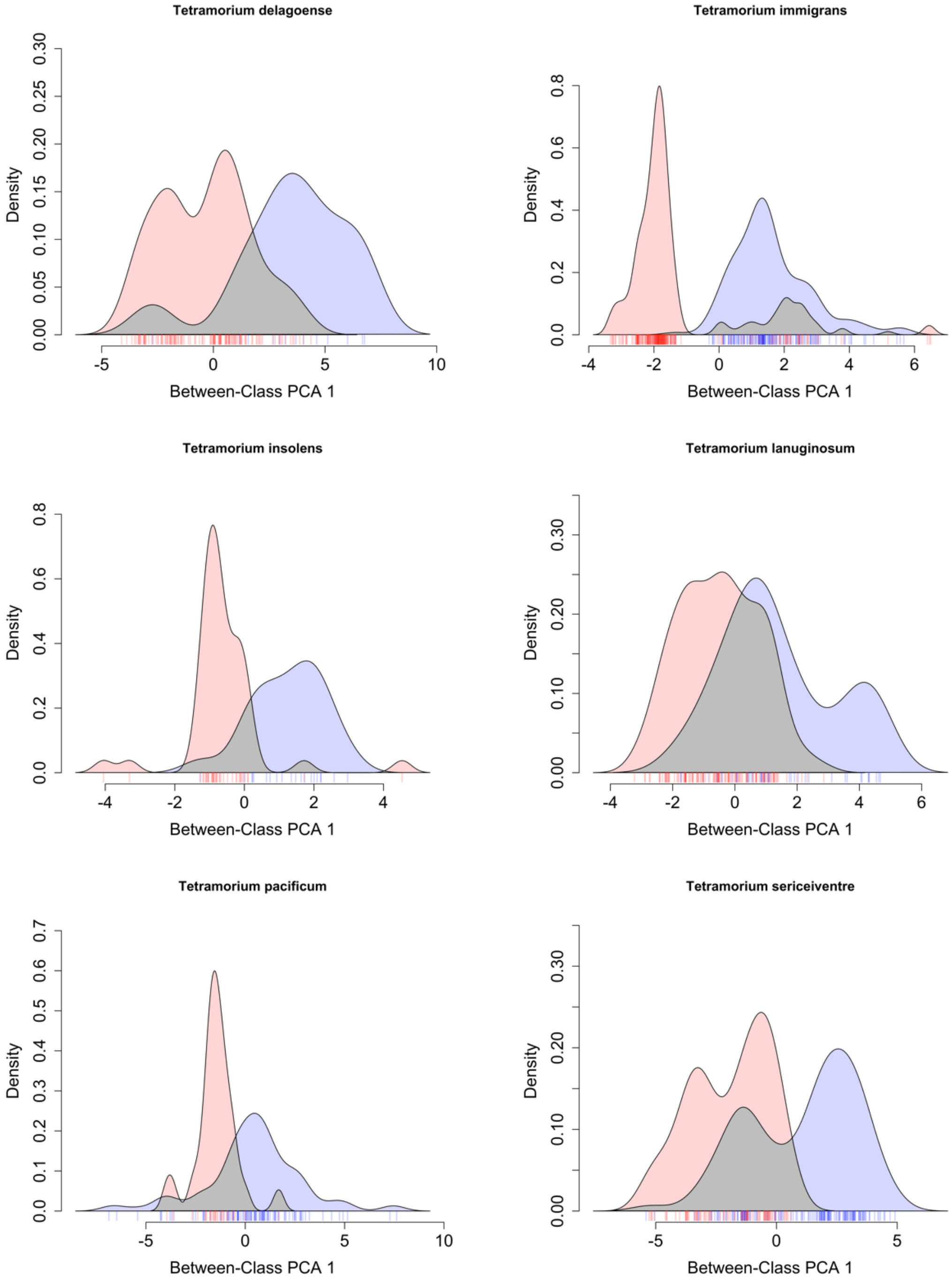
Tetramorium delagoense
Tetramorium immigrans
Tetramorium insolens
Tetramorium lanuginosum
Tetramorium pacificum
Tetramorium sericeiventre
Density
Between-Class PCA 1

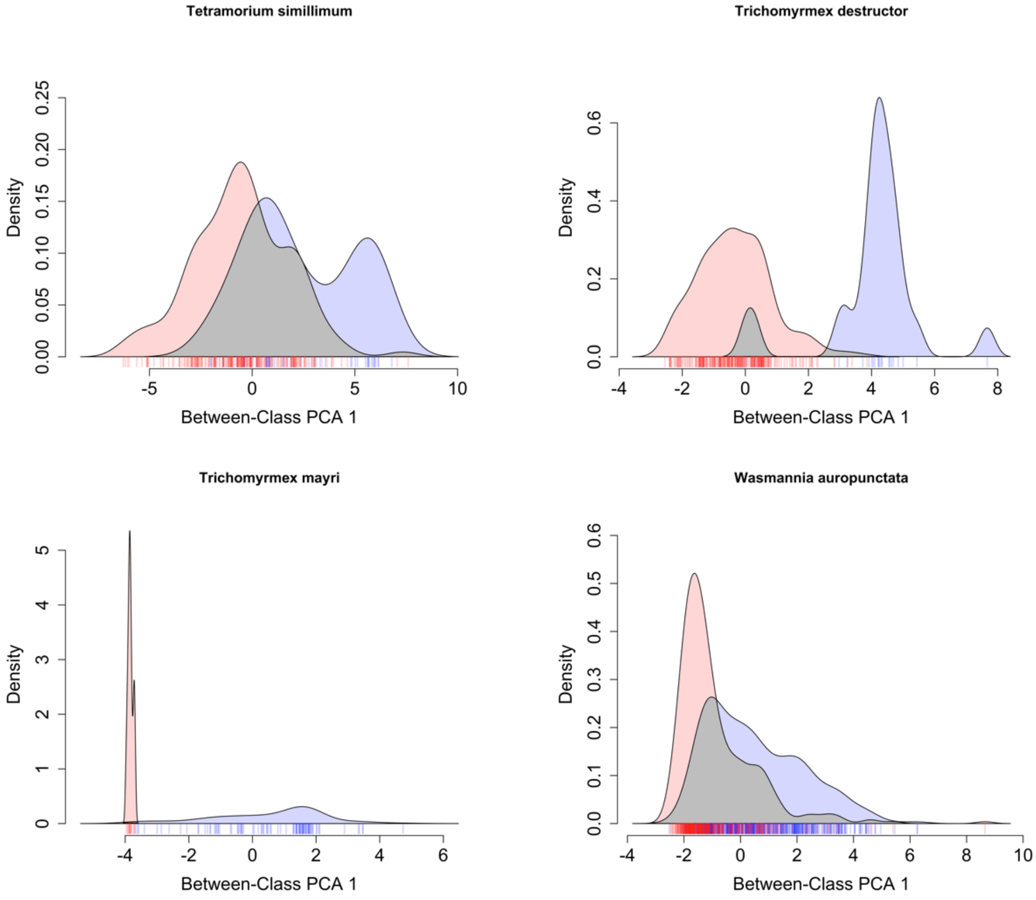
Tetramorium simillimum
Trichomyrmex destructor
Trichomyrmex mayri
Wasmannia auropunctata
Density
Between-Class PCA 1

Figure S1
