# Supplementary material for: Smaller climatic niche shifts in invasive than non-invasive alien ant species
Source: Nat Commun. 2020 Oct 15;11:5213. doi: 10.1038/s41467-020-19031-1 (PMC7567077; doi:10.1038/s41467-020-19031-1)
Supplement: Supplementary file 3 — Reporting Summary [file 41467_2020_19031_MOESM3_ESM.pdf]

nature research

Corresponding author(s): Olivia K Bates, Cleo Bertelsmeier

Last updated by author(s): Sep 22, 2020

Reporting Summary

Nature Research wishes to improve the reproducibility of the work that we publish. This form provides structure for consistency and transparency in reporting. For further information on Nature Research policies, see our Editorial Policies and the Editorial Policy Checklist.

Statistics

For all statistical analyses, confirm that the following items are present in the figure legend, table legend, main text, or Methods section.

n/a	Confirmed	
☐	☒	The exact sample size (*n*) for each experimental group/condition, given as a discrete number and unit of measurement
☒	☐	A statement on whether measurements were taken from distinct samples or whether the same sample was measured repeatedly
☐	☒	The statistical test(s) used AND whether they are one- or two-sided<br>*Only common tests should be described solely by name; describe more complex techniques in the Methods section.*
☐	☒	A description of all covariates tested
☐	☒	A description of any assumptions or corrections, such as tests of normality and adjustment for multiple comparisons
☐	☒	A full description of the statistical parameters including central tendency (e.g. means) or other basic estimates (e.g. regression coefficient) AND variation (e.g. standard deviation) or associated estimates of uncertainty (e.g. confidence intervals)
☐	☒	For null hypothesis testing, the test statistic (e.g. *F*, *t*, *r*) with confidence intervals, effect sizes, degrees of freedom and *P* value noted<br>*Give P values as exact values whenever suitable.*
☒	☐	For Bayesian analysis, information on the choice of priors and Markov chain Monte Carlo settings
☒	☐	For hierarchical and complex designs, identification of the appropriate level for tests and full reporting of outcomes
☐	☒	Estimates of effect sizes (e.g. Cohen's *d*, Pearson's *r*), indicating how they were calculated

Our web collection on statistics for biologists contains articles on many of the points above.

Software and code

Policy information about availability of computer code

Data collection	All species occurrence points were downloaded manually without computer code. All other data was downloaded within R programming, and code is available within the supplied R script on GitHub.
Data analysis	All analyses were done in R version 3.6.0 , all code for figures and data analysis is available using an R script on GitHub, which also contains all data used Github (https://github.com/OliviaKBates/AlienInvasiveNicheShift).

For manuscripts utilizing custom algorithms or software that are central to the research but not yet described in published literature, software must be made available to editors and reviewers. We strongly encourage code deposition in a community repository (e.g. GitHub). See the Nature Research guidelines for submitting code & software for further information.

Data

Policy information about availability of data

All manuscripts must include a data availability statement. This statement should provide the following information, where applicable:

- Accession codes, unique identifiers, or web links for publicly available datasets
- A list of figures that have associated raw data
- A description of any restrictions on data availability

All data used in this study can be downloaded from Github (https://github.com/OliviaKBates/AlienInvasiveNicheShift/blob/master/data_invasiveants.RData). Worldclim Global Climate Database (https://www.worldclim.org) and the AntMaps database (https://antmaps.org) can both be accessed through their respective websites.

Field-specific reporting

Please select the one below that is the best fit for your research. If you are not sure, read the appropriate sections before making your selection.

☐ Life sciences ☐ Behavioural & social sciences ☒ Ecological, evolutionary & environmental sciences

For a reference copy of the document with all sections, see nature.com/documents/nr-reporting-summary-flat.pdf

Ecological, evolutionary & environmental sciences study design

All studies must disclose on these points even when the disclosure is negative.

Study description	This study was a computational and entirely carried out in R programming. For each species, the climate niche space was determined using a between principle components analysis based on WorldClim climate data for each occurrence point, using native and non-native range as a priori groups. Following kernel density smoothing in niche-space, niche metrics were calculated using R programming to quantify the difference in niche between ranges for each species. This was done for 82 different species. All analysis afterwards was based on grouping species based on their attributes (Invasive status, observed geographical spread, degree of niche expansion calculated), using non-parametric tests.
Research sample	Data for each species was downloaded from AntMaps , which uses the Global Any Biodiversity Informatics project database. It provides specific occurrence points for each species, along with classifications of whether each occurrence point is within the native or non-native range.
Sampling strategy	All non-native ant species were considered for the study, however all those for which we had less than 10 occurrence points in either their native or non-native range were removed from the analysis, resulting in 82 species included in the study.
Data collection	All species occurrence was downloaded from the AntMaps database in 2019. All other data was calculated or downloaded using the R script provided on GitHub
Timing and spatial scale	Data was acquired in 2019, and includes all recorded occurrence points for each species to date. Climate data from WorldClim provides near-present climate data, from the years 1970-2000.
Data exclusions	All suitable data was included in the study. Species where there were less than 10 occurrence points for either their native or non-native range were removed from the study, as we determined that below this number determination of niche would be unreliable. For each of the remaining 82 species, we used the nearest neighbour distance (NND) method to thin the data, where occurrence points that were ≤0.02 units away from each other were removed (roughly 2 km) to avoid errors due to spatial autocorrelation. As the resolution of the climate maps was larger than this distance, duplicate records in the same climate grid cells were removed.
Reproducibility	All analysis is repeatable using the R script provided on GitHub.
Randomization	We quantified every variable for each species in the same way, and grouped species for analysis based on the attributes of each species (Invasive status, extent of geographical spread, extent of niche expansion)
Blinding	Blinding was not relevant to this study. All data was downloaded from suitable databases and all analysis were done using the same code for each species.

Did the study involve field work? ☐ Yes ☒ No

Reporting for specific materials, systems and methods

We require information from authors about some types of materials, experimental systems and methods used in many studies. Here, indicate whether each material, system or method listed is relevant to your study. If you are not sure if a list item applies to your research, read the appropriate section before selecting a response.

Materials & experimental systems

n/a	Involved in the study	
☒	☐	Antibodies
☒	☐	Eukaryotic cell lines
☒	☐	Palaeontology and archaeology
☒	☐	Animals and other organisms
☒	☐	Human research participants
☒	☐	Clinical data
☒	☐	Dual use research of concern

Methods

n/a	Involved in the study	
☒	☐	ChIP-seq
☒	☐	Flow cytometry
☒	☐	MRI-based neuroimaging
