# Supplementary material for: Smaller climatic niche shifts in invasive than non-invasive alien ant species
Source: Nat Commun. 2020 Oct 15;11:5213. doi: 10.1038/s41467-020-19031-1 (PMC7567077; doi:10.1038/s41467-020-19031-1)
Supplement: Supplementary file 4 — Description of Additional Supplementary Files [file 41467_2020_19031_MOESM4_ESM.pdf]

Description of Additional Supplementary Files

File Name: Supplementary Data 1
Description: Individual results for each species used in the study. Raw values are given for metric values and number of occurrence points for each species. Invasive status (A=Alien, I=Invasive) is as categorised by the IUCN. All the per-species overlap graphs.
